# Energetic trade-offs: Implications for selection between two bivalve prey species by a carnivorous muricid gastropod

**DOI:** 10.1371/journal.pone.0250937

**Published:** 2021-04-30

**Authors:** A. Averbuj, J. A. Büchner-Miranda, L. P. Salas-Yanquin, J. M. Navarro, L. M. Pardo, A. S. Matos, J. A. Pechenik, O. R. Chaparro

**Affiliations:** 1 Laboratorio de Reproducción y Biología Integrativa de Invertebrados Marinos (LARBIM)–IBIOMAR, CCT CONICET–CENPAT, Puerto Madryn, Chubut, Argentina; 2 Instituto de Ciencias Marinas y Limnológicas, Universidad Austral de Chile, Valdivia, Chile; 3 Centro FONDAP de Investigación de Dinámicas de Ecosistemas Marinos de Altas Latitudes (IDEAL), Valdivia, Chile; 4 Departamento de Biologia, Centro de Ciências, Laboratório de Invertebrados Marinhos do Ceará, Universidade Federal do Ceará, Fortaleza, Brasil; 5 Biology Department, Tufts University, Medford, MA, United States of America; University of California, UNITED STATES

## Abstract

Active predators obtain energy and nutrients from prey through complex processes in which the energy gained must exceed the energy invested in finding and ingesting the prey. In addition, the amount of energy available will vary with the prey that are selected for consumption. The muricid gastropod *Acanthina monodon* inhabits rocky shores, where it routinely feeds on the mytilids *Semimytilus algosus* and *Perumytilus purpuratus*. In this study, *S*. *algosus* was highly preferred by the predator (over 90% were eaten) versus *P*. *purpuratus* (only 9% were eaten) when offered a mixed diet. The energetic cost of attacking one *S*. *algosus* individual was 91 J bivalve^-1^ while for *P*. *purpuratus* it was slightly higher: 95 J bivalve^-1^. Also, whereas *A*. *monodon* required on average 19 h to consume *S*. *algosus*, successful attacks on *P*. *purpuratus* required about 32% more time (25 h). In addition, a longer resting time was needed by the predator after preying on *P*. *purpuratus* before it initiated another attack. Moreover, the active metabolic costs associated with successfully attacking the prey increased 3.2 times over the basal metabolic costs when attacking *S*. *algosus*, but only by 2.5 times when attacking *P*. *purpuratus*. The calculations associated with preying on each species showed that the energetic gain per unit time likely accounts for the predator’s preference for attacking *S*. *algosus*, even though predation on both species provided net energy gains for the predator. However, as *S*. *algosus* occurs seasonally at our study site, *P*. *purpuratus* would probably also be consumed due to its constant availability throughout the whole year.

## Introduction

The search for food by heterotrophic organisms may have a high energetic cost. Prey availability may determine predator species distributions and even force ontogenetic shifts in prey selection by the predator, in particular if prey density is low [1, 2]. It is mostly accepted that consumers select their food in order to maximize their rates of net energy gain, i.e. reducing finding and manipulation costs and/or by accessing more energy-rich prey [3]. Energy gain is not the only influencing factor, as there are also different important nutrients that are present in food sources, or in certain organs that are selectively ingested [4–6].

The energetic costs of the feeding process become higher if prey present protective structures (e.g. shell valves or carapaces) that should be penetrated in order to consume their soft tissues [6]. Larger predators can select prey that offer a better nutrient source and also higher chances of being successfully attacked [7–9]. When different prey species are available that are equal in their energy and nutritional content, prey availability and encounter rate are expected to determine predator preference [6]. Non-specific predators commonly consume organisms that differ in size, shape, soft tissues content, and the hardness and thickness of protective structures; thus, one or more of these parameters may define the selection of one prey over another. This is probably related to a net balance between the energetic costs of attack and the energetic income from consuming a particular prey [6, 7, 10, 11].

Thus, searching for food, attacking and manipulating prey, and/or the ingestion-digestion processes themselves, may be largely responsible for increasing the energetic costs associated with carnivory [12–14], and are all considered in general as active metabolism. During feeding by some predator species, it is hard to distinguish between one phase and a following phase, as the feeding activity appears as a single, continuous process [15]. Nonetheless, the increment of feeding costs is sustainable and represents a benefit for the predator when total feeding costs (all phases added together) are minor compared with the energetic incomes obtained from the consumed prey [10, 11].

Carnivorous gastropods typically feed on encrusting organisms such as sponges, barnacles, or tunicates, but they may also consume echinoderms and bivalve molluscs [16–20]. When dealing with bivalves, gastropod predators are forced to deal with a diversity of shell valve shapes and hardness that protect the prey; these differences could become important determinants in prey selection by the predatory gastropod. Besides the likely relevance of the difficulty in penetrating the shell valves of the prey and the time that must be invested in the process and the associated energetic costs, the potential risk assumed by the predator (e.g. being attacked by other top predators while finding and/or consuming their prey) could also be an important determinant for prey selection [21–23].

*Acanthina monodon* (Pallas 1774) is a muricid gastropod that inhabits most rocky shores along the Chilean coast [24–26]. Although this species may also include barnacles in its diet, over 95% of its consumed prey are mytilid bivalves [17], which it obtains by first cracking the bivalve’s shell [27]; the sympatric mussels *Semimytilus algosus* and *Perumytilus purpuratus* are the most commonly consumed species by *A*. *monodon* along the Chilean southern coasts. However, *S*. *algosus* is more frequently consumed than *P*. *purpuratus* [17], possibly because the shell valves of *S*. *algosus* are thinner and easier to penetrate [17, 27]. *Acanthina monodon* uses four different mechanisms to prey on these two mytilid species: ABO (accesory boring organ), radula, labral tooth, and the pedal muscle, and their relative roles of each are variable depending on the ontogenic development of the gastropods [27, 28]. In our study, we proposed that prey selection is not only influenced by the energetic costs involved in the attack process, but also with the degree to which energy intake obtained from the prey overcomes those costs; in other words, selection should favor net energy gained in prey selection. In the present study, we considered the energetic content of the prey, the time invested in attacking and consuming the prey, the force required to break the prey’s shell valves to access the tissue for ingestion, the different resting times between two consecutive predatory events for both prey species, and the potential roles of each of these factors in determining prey selection by *A*. *monodon*.

## Materials and methods

### Collecting animals and maintaining them in the laboratory

Individuals of the carnivorous gastropod *Acanthina monodon* were collected from the rocky intertidal zone along Calfuco beach, Chile (39° 79´27´´S; 73° 39´27´´W) between January and March (summer, Southern hemisphere) of 2019. Simultaneously, the mytilids *Semimytilus algosus* and *Perumytilus purpuratus* were collected at the same field site, to be offered as prey. All collected individuals were taken to the nearby Calfuco research laboratory and maintained in filtered (to 0.5 μm) flow seawater obtained directly from where the animals had been collected; salinity was 31.4 ± 0.6 psu, water temperature was 12.5° C ± 0.5. The seawater in each aquarium was aerated constantly. The gastropods and mussels were collected and brought immediately and directly to the lab, which is less than 100m away from where they were collected. Also, individuals were always maintained submerged in seawater taken from the same area, to minimize manipulation stress that might affect their feeding behavior. The captured gastropods were kept in tanks for no longer than 48 h before experiments were carried out.

Before starting the experiments, all epibionts were carefully removed from the shells of all individuals (both predator and prey), to avoid the possibility that epibionts might interfere with prey selection or prey capture, as well as in quantification of oxygen consumption rates (OCR).

### Prey selection and attack rate

A total of 75 transparent containers (500 ml each) were placed inside six 50 L aquaria. Each of the treatments (e.g. *P*. *purpuratus* monodiet, *S*. *algosus* monodiet, or a mixed diet of both mussel species) were maintained in two separated 50 L tanks, to avoid mixing the prey-attracting chemical signals originating from each prey species. Several small holes (surface area 0.5 cm^2^ each were drilled in each container to enable water circulation from the main aquaria to the interior where the gastropods and their prey were placed.

The gastropods in 25 of the 75 containers were fed exclusively on the bivalve *Perumytilus purpuratus* and individuals in another 25 containers were fed exclusively on *Semimytilus algosus* (monodiets), while those in the remaining 25 containers received a mixed diet containing equal numbers of both mussel species (“mixed diet” treatment). In the two single-diet experiments, one gastropod was placed with 15 mussel prey of the appropriate species, while in the mixed diet experiments each predatory gastropod was provided with 10 *P*. *purpuratus* individuals and 10 *S*. *algosus* individuals. The mean shell length of the predatory gastropods used in this study was 28 ± 2 mm (± SD), while the mytilid prey measured 18 ± 3 mm (mean ± SD) in shell length. The size range of the prey corresponded to that of the individuals that had been consumed by the predator in previous experiments [27]. For all experiments, the mussels in each aquarium were fed (ad libitum, daily) with pure cultures of the microalga *Isochrysis galbana*.

Every 3 days, all empty mussel shells were collected from each container to determine the number of individuals that had been consumed by each predator; they were then replaced by other bivalves of similar size that had been collected from the same location. The experiments were run for 16 days. The attack rate was estimated as the number of prey consumed per day by each predatory gastropod.

### Determining rates of soft tissue ingestion by the predator

Individuals of *S*. *algosus* and *P*. *purpuratus* were sampled simultaneously from the low intertidal of Calfuco beach. Approximately 30 specimens of each species were collected, representing the range of sizes found in those populations (*S*. *algosus*: 10.7–33.9 mm SL; *P*. *purpuratus*: 5.0–30.0 mm SL). After measuring the shell length, soft tissues were separated from the shell valves, deposited in pre-weighed aluminum foil plates, dried at 60°C for approximately 48 h, and re-weighed to determine dry tissue weight. The relationship between shell length and dry tissue weight was then used to estimate the tissue weights of individuals that were consumed in the experiments, based on their shell lengths.

Ingestion rates were expressed as the amount of dried soft tissue consumed by the predator *A*. *monodon* per day.

### Estimating attack-ingestion times and resting time between periods of prey consumption

A total of 120 gastropods of 28 ± 2 mm SL, were placed into four 50 L aquaria, with 30 individuals per tank. One hundred individuals of *S*. *algosus* were added to each of two tanks, and 100 individuals of *P*. *purpuratus* were added to each the two other tanks (30 predators:100 prey). The mytilid prey measured 18 ± 3 mm (mean ± SD) in shell length. All of the experimental aquaria were filled with seawater taken directly from where animals had been collected, with a salinity of 31.4 ± 0.6, a temperature of 12.5 ± 0.5°C, and constant aeration. In each tank, half of the seawater was changed every two days, taking care that the animals always remained submerged. A digital video camera was installed over each aquarium to continuously record the feeding activity over a 10-day experimental period. A ratio of 1 gastropod: 3.3 prey was maintained. Every 3 days, the valves of consumed prey were collected and replaced immediately with the same number of mussels of similar sizes. Video analyses allowed us to estimate the time taken by the gastropods to complete each attack and to calculate an average attack-ingestion time. This attack-ingestion time was calculated from the moment that the snail was positioned over the prey until the prey was abandoned.

Resting time was estimated from the attack-ingestion monodiet and mixed diet experiments, over a 10-day period. The difference between the total 10-day period and the effective total attack/ingestion time (taking into account the total number of consumed prey and the mean attack time needed per prey), was considered as the resting time. At this stage, it was not possible to distinguish between resting time (associated with the digestion and absorption of prey) and time spent searching for prey, due to the predator behavior: after feeding, the predators presented periods of total resting mixed with periods of mobile activity not associated with prey searching activity. Thus, in this study the complete period between two consecutive attacks was considered as resting time. This resting time was later used to estimate basal metabolic costs and for the later energetic profit calculations.

### Oxygen consumption rate and energetic costs

The oxygen consumption rates (OCR) (mg O_2_ h^-1^ ind^-1^) for gastropods that were resting (basal metabolic rates) or were actively attacking and ingesting prey (active metabolic rates) were estimated following the methodology described by Salas-Yanquin et al. [29].

Basal activity measurements were obtained 24 to 80 h after the gastropods had consumed one of the prey species. For the “active phase” measurements, OCR’s were determined at different stages of the feeding process (including attack and ingestion, but not distinguishing between them). Thus, OCR was estimated covering all phases of the predator feeding process [30].

In order to estimate active OCR, mussels (*S*. *algosus* or *P*. *purpuratus)* were first allowed to attach with their byssal threads to a small gravel rock to facilitate manipulation during the experiment. Once the carnivorous gastropod was placed over the prey, the whole system (rock + prey + predator) was moved inside the respirometry chamber (without touching the animals), and then the chamber was hermetically sealed under water. The effective volume of seawater in the chamber varied between 140 and 800 ml (not considering the system rock + prey + predator). The seawater (salinity of 31.8 ± 0.6 psu, mean ± SD) used to fill the chambers was previously filtered and UV sterilized and maintained at a temperature of 15 ± 0.72°C. The seawater was well-aerated before use, but aeration was turned off 15 minutes before an experiment was initiated, to allow mini-bubbles to be eliminated. The OCR was measured for each gastropod for 1 hour. The whole system (rock + prey + predator) was then returned into an aerated tank of seawater; the chamber containing the animals was opened under water, and remained there for 1–2 h, after which the OCR measuring process was repeated. After closing the respirometry chamber, we waited for 5 min before starting a new oxygen concentration measurement, to avoid possible influence of chamber manipulation. The number of measurements ranged between 1 and 4 for each gastropod, depending on when the OCR of the attacking snail was measured for the first time. During measurements, the animals were always submerged in seawater and were never exposed to air. Oxygen concentrations were determined non-invasively, using a Fibox 3 oximeter (Precision Sensing gmbH) with optical Spot SP-PSt3-NAU sensors. Concurrently, OCR’s were determined for two control respirometry chambers, one with a rock without animals and the other only with seawater. After the last OCR measurement was taken for each gastropod, the total seawater volume (excluding the rock and animals) in each chamber was measured.

The OCR’s of all gastropods obtained at different times during the attack-ingestion process were averaged according the consumed prey species. The total energy cost of the active phase considered the duration of the attack and ingestion period, according to the attacked prey species. The mean OCR values corresponding to each attack event (and for each prey species) were then transformed to energy consumption values using the conversion factor of 1 ml O_2_ consumed = 19.9 J [31]. For energy consumption estimations, the OCR of predators was considered similar during the whole attack period, due to the difficulty of identifying phases included throughout the attack-ingestion process. In all calculations, the declines in oxygen content within the respirometry chambers were assumed to reflect the gastropods’ respiratory activity, as the bivalve prey close their valves tightly [32] during an attack (B. Riedemann, Pers. Comm.,), which would prevent them from consuming oxygen from the surrounding seawater.

### Prey caloric content

Ten individuals of each prey species were sampled during January from Calfuco beach. After measuring their maximal shell length, the soft tissues were completely removed, dipped quickly in distilled water to remove adhering salts, and placed in pre-weighed aluminum foil plates. Samples were then dried for 48 h to 60°C, until weights had stabilized, and were then re-weighed to the nearest 0.1 mg, to obtain the mean dried tissue weight per individual for both mussel species. The range of prey sizes used for the weigh determinations were the same as those used in the prey selection experiments (see *Prey selection and attack rate*, 1.8 ± 0.3 cm). The dry tissues of both prey species were ground using a mortar and pestle and then homogenized for determinations of caloric content.

Dried and ground samples were made into pellets with a Parr 2812 press, for calorimetric determinations. A digital top balance was used to determine sample weights to the nearest 0.1 mg prior to their ignition in a Parr 1109a microcalorimeter to obtain the energetic content of each prey individual. Calibrations were periodically carried out using benzoic acid [33]. The values obtained were corrected for ash and acid content and expressed as KJ/g ash-free dry weight (AFDW) to obtain the energy density (ED). The energy content (EC) of soft tissues was calculated as EC = ED * DST.

### Energetic summary

The energetic profit gained by *A*. *monodon* through predation was estimated considering both the costs associated with the basal metabolism of the gastropods as well as their active metabolism during the attack and ingestion of prey (*S*. *algosus* or *P*. *purpuratus* fed both monodiets or mixed diets). These costs were estimated for the attack and resting times corresponding to each prey species, considering also the ingestion rate and the energetic content of the biomass consumed. The energy estimations were calculated per predatory gastropod based on a 10-day experimental period.

Spatial distribution of the attacks on prey valves and compressive force required to break prey valves.

The shell valves of all mussels consumed in the prey selection experiments were collected and photographed. The spatial distribution of the predator’ attacks on the collected shells were identified using a stereomicroscope. These recorded images were later plotted over a model bivalve figure.

To assess the forces required to break the shells of living bivalves of both species, individuals of *P*. *purpuratus* (n = 76 individuals; 6 to 32 mm SL) and *S*. *algosus* (n = 120 individuals; 9 to 33 mm SL) were collected from the intertidal rocky shore at Calfuco beach. The compressive force required to break their shells was then determined by applying an increasing force using a PCE Instrument Force Gauge (model PCE-FM50) on the outer side of the right valve. The force was applied until the shell valve cracked on one of each of four shell valve areas: area 1 (center of the shell valve); area 2 (anterior of the shell valve, umbo area); area 3 (ventral-central side of the shell valve); area 4 (ventral posterior side of the shell valve) [34]. The force needed to break the shell valves of both mytilid species was quantified using a dynamometer with a maximum capacity of 54 Newton (circa 5400 g force).

### Statistical analyses

Normality and homoscedasticity of the data were tested with Kolmogorov-Smirnov and Levene tests, respectively. When such criteria were not satisfied, non-parametric tests were used to analyze the data.

One way-ANOVA was used to test for differences among attack rate and consumption rate between both single-species diets. A non-parametric Mann-Whitney test was used to determine differences for the attack and ingestion rates of the predator on the two different prey species when the predators were offered the mixed diet. ANCOVA analyses were used to test for differences of the required force to break the valves of *P*. *purpuratus* and *S*. *algosus*, between species and between valve areas of each species (4 different areas), using prey size as a covariate.

A t-test was used to compare the attack-ingestion times of *A*. *monodon* preying on *P*. *purpuratus* and *S*. *algosus*, as well as for comparing the energetic content of both prey species. Differences in mean oxygen consumption rates of *A*. *monodon* preying on both bivalve species were tested with a non-parametric Mann-Whitney test.

In all tests, *P* < 0.05 was considered to be statistically significant. Statistical analyses were carried out by using STATISTICA program version 7.0.

### Ethics statement

The three species involved in this research are not endangered or protected; therefore, no specific permissions were required to sample or remove individuals from this location.

## Results

### Prey selection and attack rates

**Fig 1:**
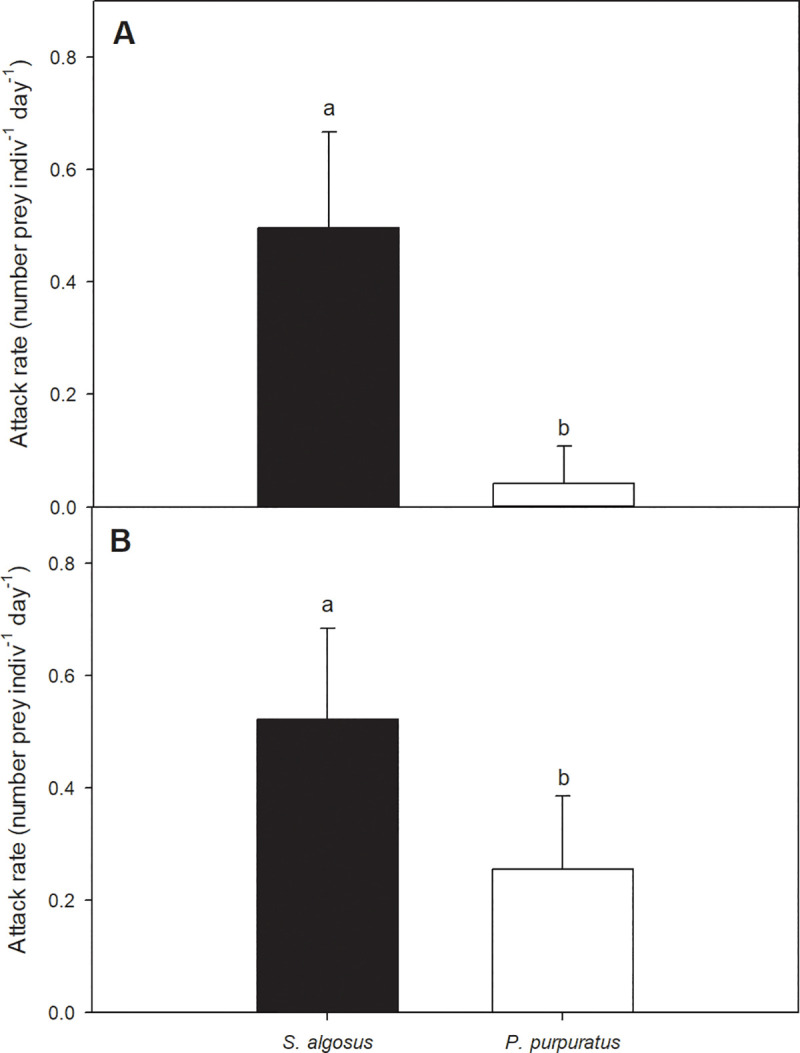
Prey preference of the predator *A*. *monodon* between *S*. *algosus* and *P*. *purpuratus*. A) Number of prey attacked per day by the predatory gastropod *A*. *monodon* (mean ± SD) when the gastropod was offered a mixed diet; Mann-Whitney: p < 0.0001; Z = 8.53; n = 100. B) Number of prey attacked per day (mean ± SD) when the predator was offered a monodiet; one-way ANOVA: p < 0.0001; F_(1, 52)_ = 43.1; n = 54.

When exposed to a mixed diet, *A*. *monodon* almost always showed a preference for attacking *S*. *algosus* (91% of the predatory events) compared to *P*. *purpuratus* (Fig 1A, Mann-Whitney: p < 0.0001; Z = 8.53). Even when the two prey species were offered separately, in monodiet experiments, *S*. *algosus* was attacked significantly more often than *P*. *purpuratus* was attacked (Fig 1B; One way-way ANOVA: *P* < 0.0001; F_(1, 52)_ = 43.1), although the differences were less dramatic (*P*. *purpuratus* was attacked 52% less than *S*. *algosus*).

### Soft tissue content of prey and ingestion rate of the predator

**Fig 2:**
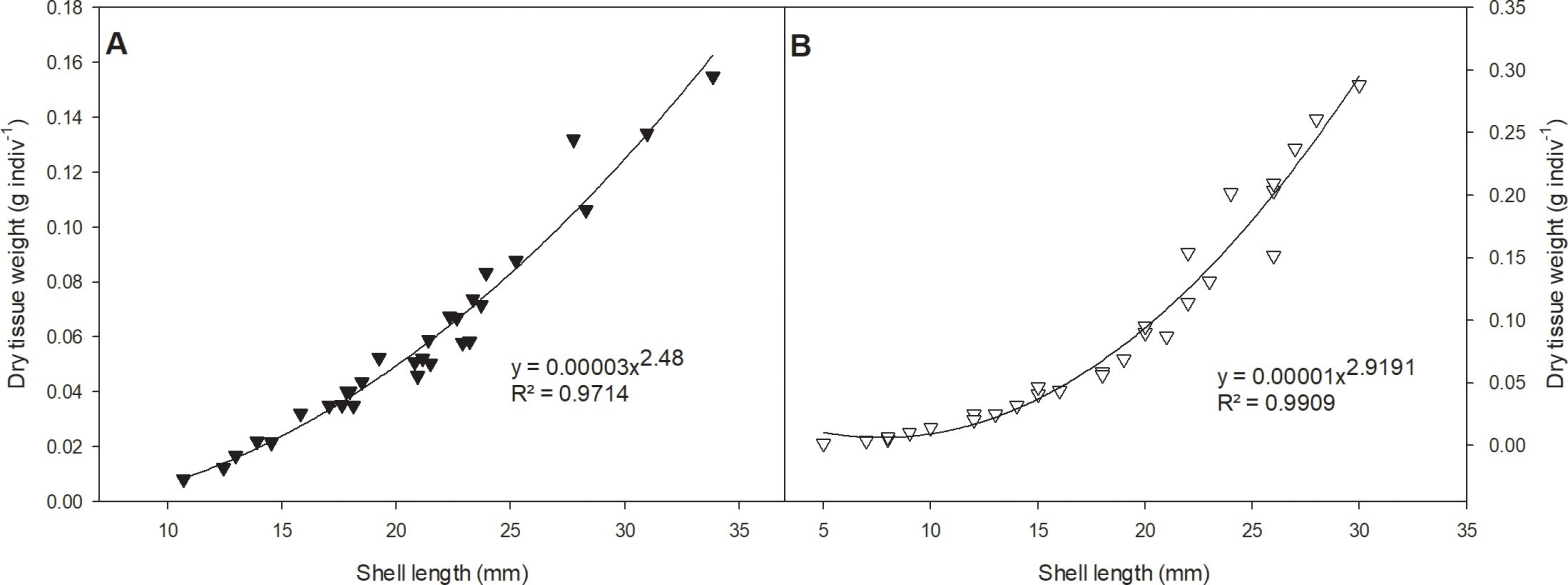
Relationship between dry tissue content and shell length of both prey species. A) *S*. *algosus* (n = 30) and B) *P*. *purpuratus* (n = 30).

The tight relationship established between shell length (mm) and biomass (g of dry weight) in the mussels *S*. *algosus* (y = 0.00003X^2.48^, R2 = 0.9714) and *P*. *purpuratus* (y = 0.00001X^2.9191^, R2 = 0.9909) enabled us to estimate the biomass of prey (based on the sizes of the recovered empty shell valves) that were consumed in the experiments (Fig 2A and 2B).

**Fig 3:**
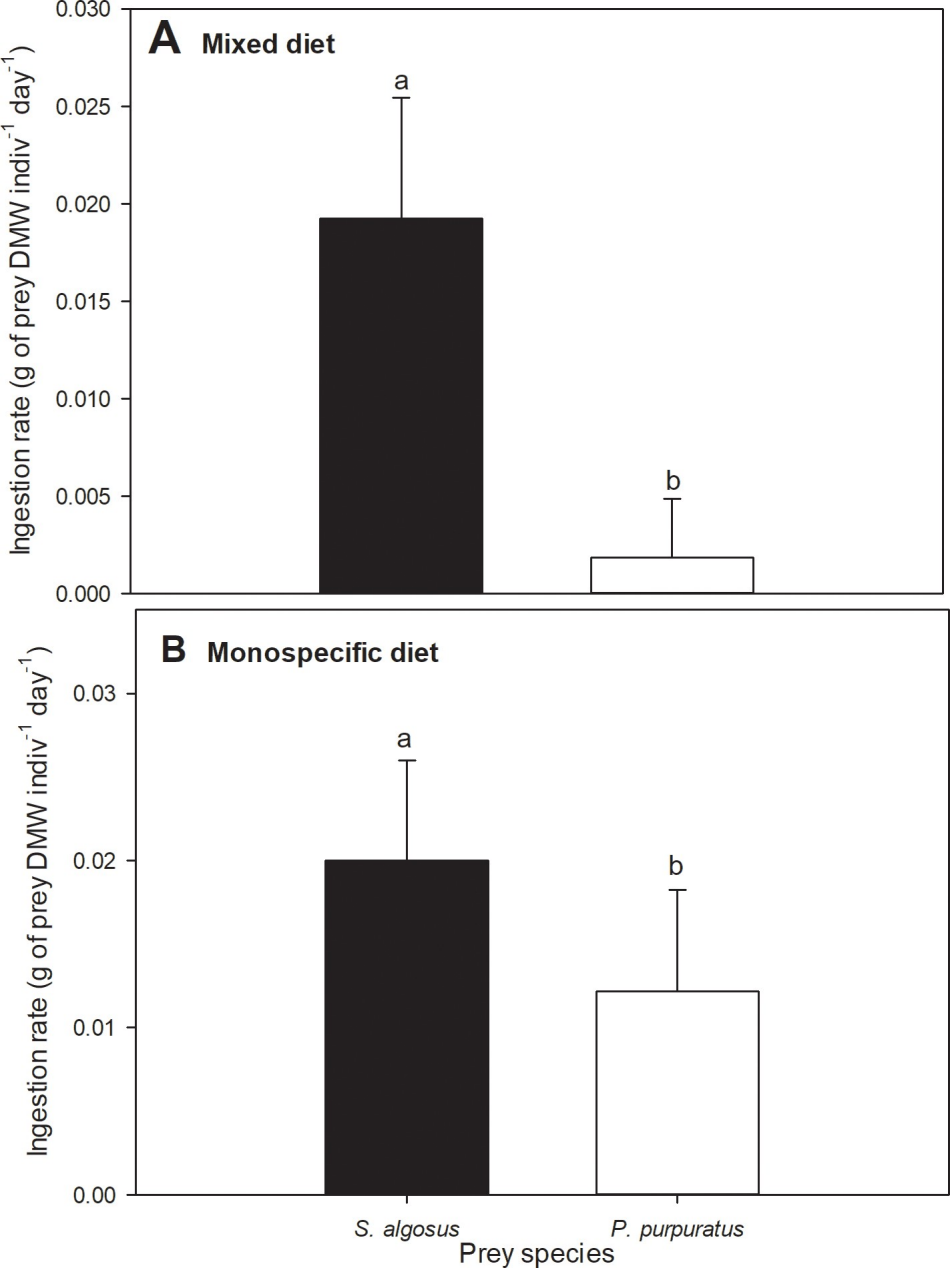
Ingestion rate of both prey species. A) Dry tissue of the prey consumed by *A*. *monodon* per day (mean ± SD) when fed with a mixed diet; n total = 100 (Mann-Whitney: p < 0.0001; Z = 8.51). B) Dry tissue of the prey consumed by *A*. *monodon* per day (mean ± SD) when fed with monodiets (prey species: *S*. *algosus* or *P*. *purpuratus*); n total = 54 (One-way ANOVA: p < 0.0001; F_(1, 52)_ = 22.2). Different letters over the bars indicate statistically significant differences.

Gastropods that were offered a mixed diet ingested more than 10 times as much biomass of *S*. *algosus* per day (0.019 g dry soft tissues indiv^-1^ day^-1^) as that of *P*. *purpuratus* (0.0018 g dry soft tissues indiv^-1^ day^-1^) (Fig 3A; Mann-Whitney: *P* < 0.0001; Z = 8.51).

Moreover, when the two prey species were offered separately, the mean ingestion rates on the two prey species were also significantly different (Fig 3B; one way-ANOVA: *P* < 0.0001; F_(1, 52)_ = 22.2), with *A*. *monodon* consuming 0.020 g dry soft tissues of *S*. *algosus* indiv^-1^ day^-1^ and only 0.012 g dry soft tissues of *P*. *purpuratus* indiv^-1^ day^-1^, only about 60% of the *S*. *algosus* tissue that was ingested.

### Attack-ingestion time and resting time between prey consumption

**Fig 4:**
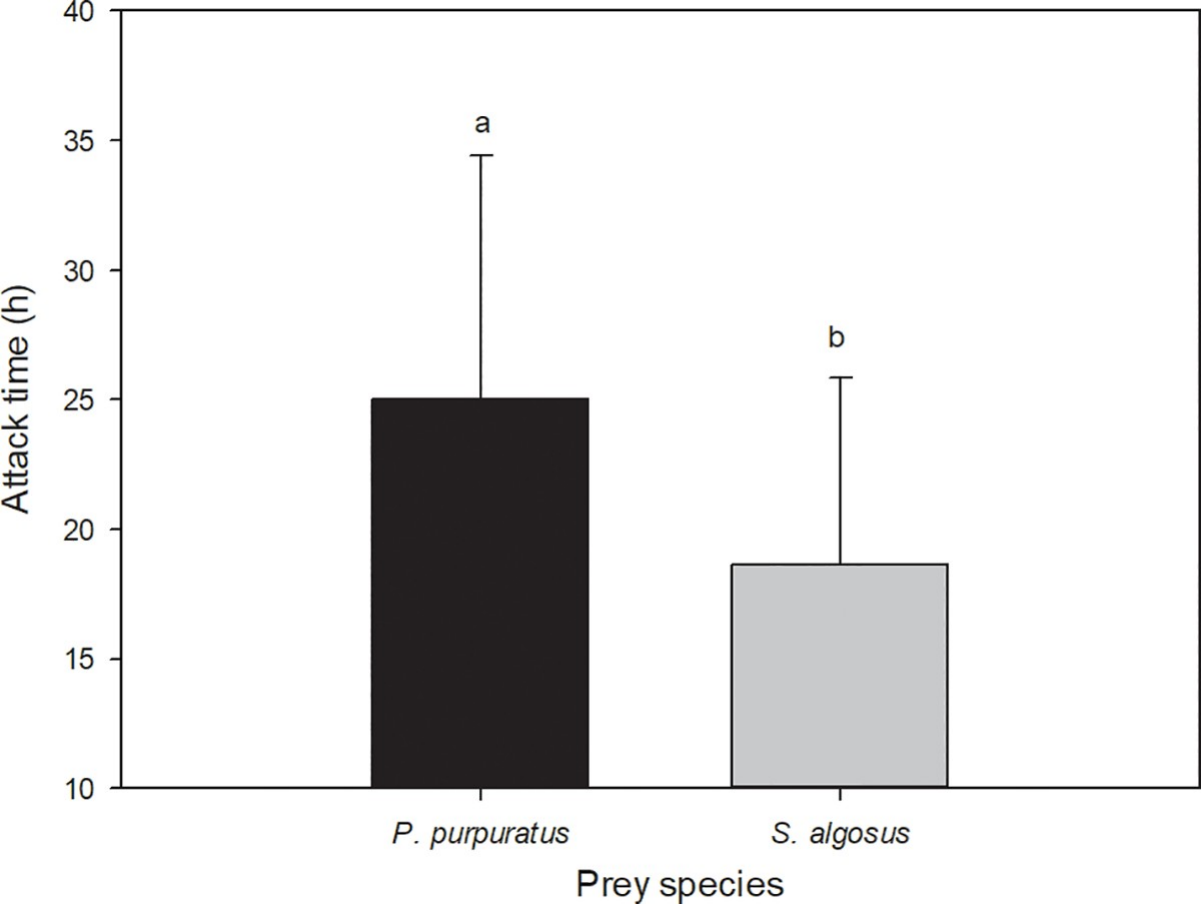
Mean time invested by *A*. *monodon* during attack-ingestion of single individuals of *P*. *purpuratus* (n = 20) and *S*. *algosus* (n = 17).

On average, *A*. *monodon* took 26% longer to attack and ingest *P*. *purpuratus* than to attack and ingest *S*. *algosus* (*t*-test value = 2.2984, *P* = 0.027; n = 37) (Fig 4).

Estimations of total resting time (time that the predators did not spend energy on attacking and eating) of *A*. *monodon*, obtained over the10-days experimental period, preying on *S*. *algosus* was 143 h, and when preying on *P*. *purpuratus* was 176 h, approximately 19% greater. Meanwhile, the total resting time of *A*. *monodon* when exposed to a mixed diet of *S*. *algosus* and *P*. *purpuratus* was 137 h over the same 10-day period.

### Oxygen consumption rates (OCR) and energetic costs

**Fig 5:**
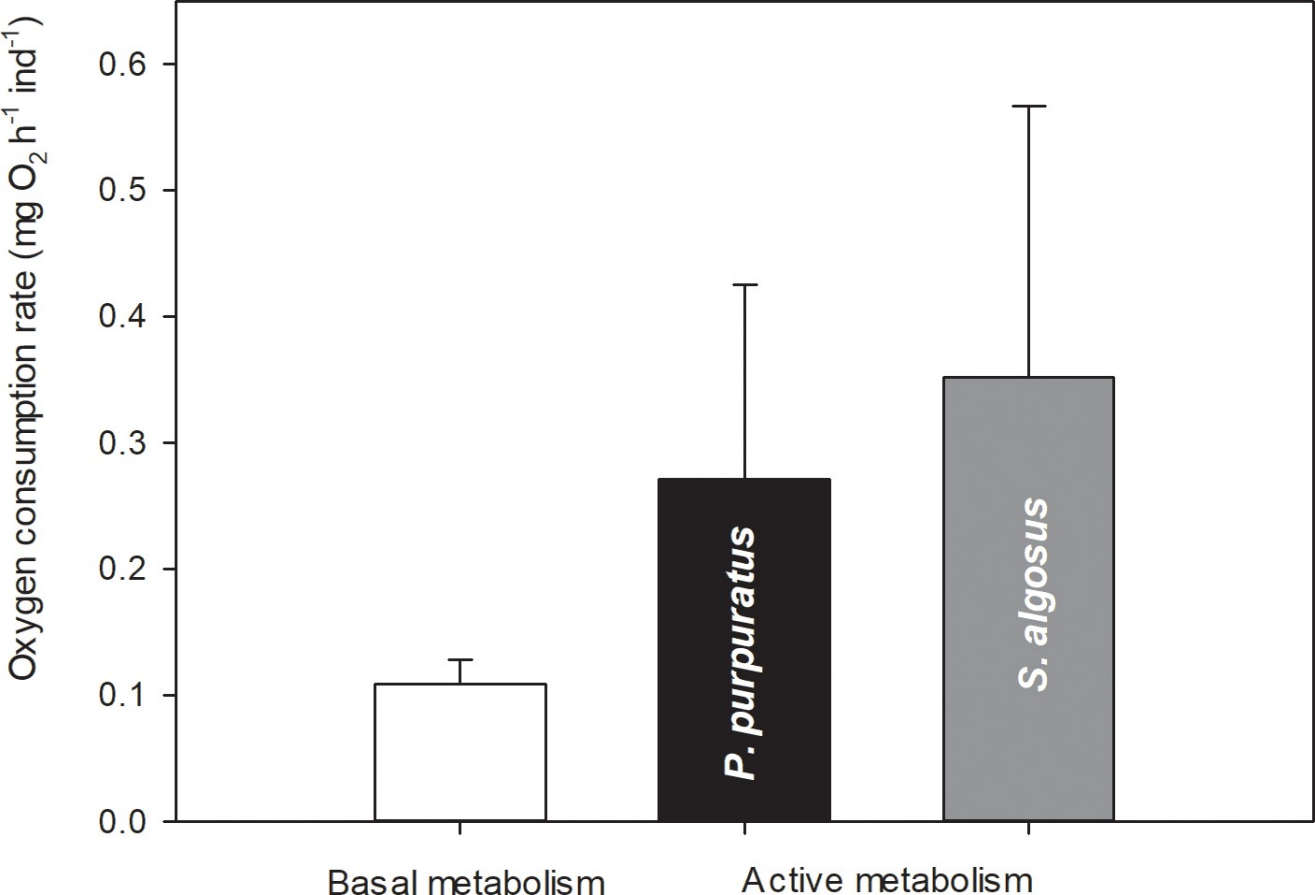
Average Oxygen Consumption Rate (OCR) of resting *A*. *monodon* individuals (basal metabolism, n = 5) and during the whole attack/ingestion process (active metabolism) of a prey specimen of *P*. *purpuratus* (n = 42) or *S*. *algosus* (n = 80).

**Fig 6:**
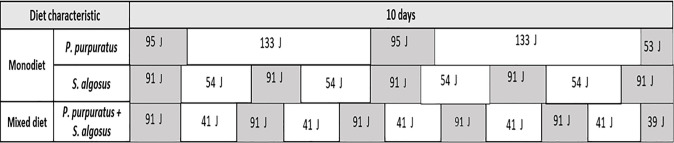
Scheme of average distribution of energy invested by each predatory gastropod during basal (white box, time between two consecutive attacks) and active (grey box, values estimated during the attack/ingestion periods) metabolism depending of diets (monodiets or mixed diet), in a 10-days experimental period.

The basal OCR estimated for resting individuals of *A*. *monodon* (i.e., for the periods between consecutive attacks) was on average 0.1089 mg O_2_ h^-1^ indiv^-1^, whereas the OCR estimated for these gastropods during periods of attack and ingestion of prey was more than 3X higher (0.3520 mg O_2_ h^-1^ animal^-1^) when they were consuming *S*. *algosus* and about 2.5X higher (0.2715 mg O_2_ h^-1^ animal^-1^) when they were consuming *P*. *purpuratus*, respectively. However, although the estimated OCR was 23% higher when consuming *S*. *algosus* than when consuming *P*. *purpuratus*, the differences between these active OCR values were not statistically significant (U Statistic = 1393.0, *P* = 0.123, n = 122), (Figs 5 and 6).

During resting periods, the mean energy invested in basal metabolism of *A*. *monodon* between two consecutive attacks when offered a monodiet was 133 J (= a mean resting time: 88h) and 54 J (= a mean resting time: 36h) for *P*. *purpuratus* and *S*. *algosus* monodiets, respectively. Meanwhile, when offered a mixed diet, the basal metabolic energy investment of *A*. *monodon* was 41 J (= a mean resting time: 27 h) (Figs 5 and 6).

### Prey energetic content

**Fig 7:**
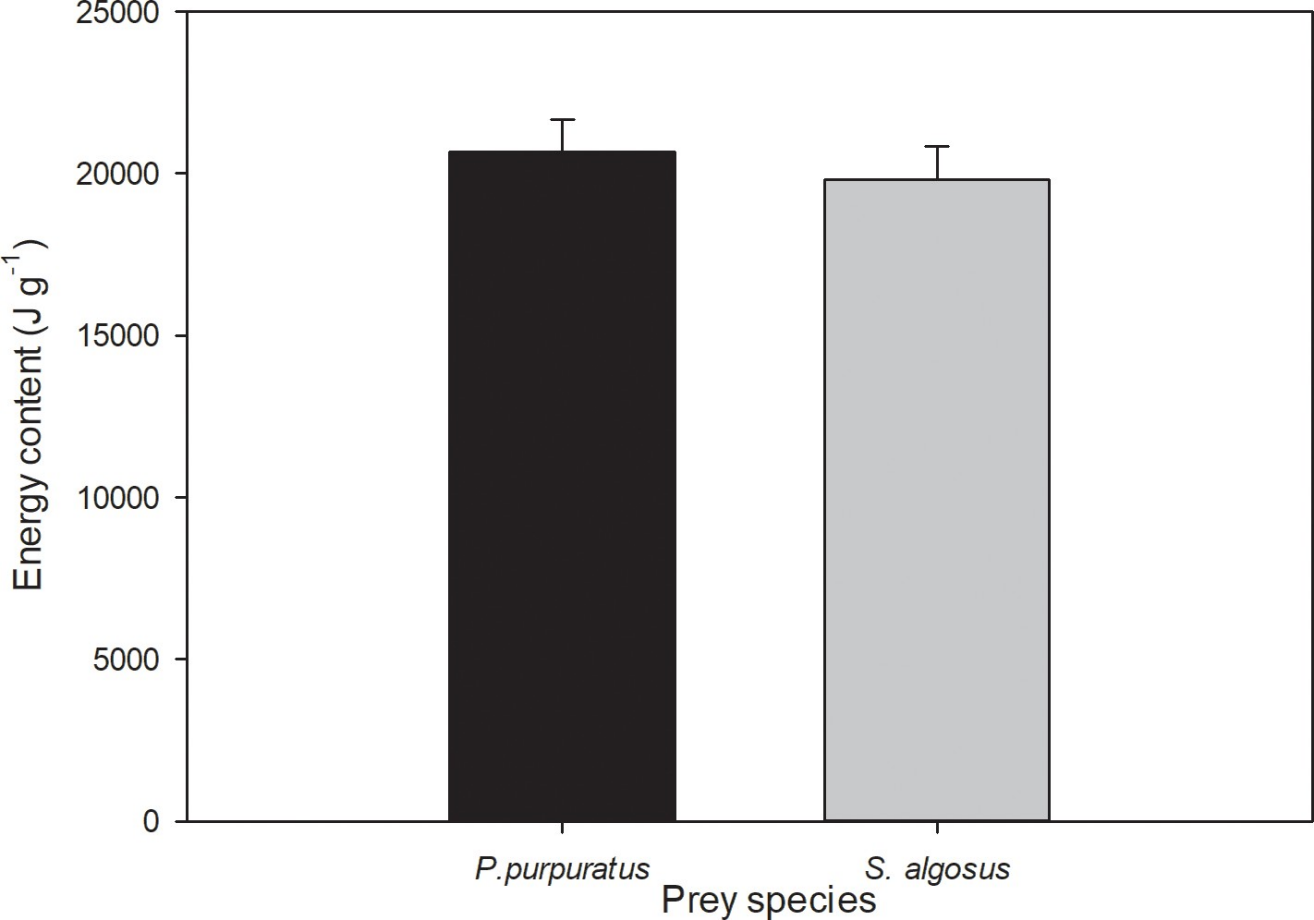
Mean energetic content (mean ± SD) of the prey soft tissues: *P*. *purpuratus* (n = 6) and *S*. *algosus* (n = 8).

The tissues of the two prey species showed no significant difference in mean energy content (*P*. *purpuratus*: 20,818 ± 1,009 J g^-1^, *S*. *algosus*: 19,812 ± 1,021 J g^-1^) (t-test value = 1.658, *P* = 0.121; n = 15, Fig 7).

### Energetic summary

#### Energetic costs

The energetic investment of *A*. *monodon* in basal metabolism (resting OCR) was associated with the duration of ´resting time´ between two consecutive attacks, which varied with the species of prey that was attacked. The average basal metabolic cost was 1.51 J h^-1^ indiv^-1^, which implies a total investment for the 10-day experimental period of 266 J indiv^-1^ (176 h of basal activity) and 216 J indiv^-1^ (143 h) when preying on *P*. *purpuratus* and *S*. *algosus* monodiets, respectively. When the gastropods were offered a mixed diet, the average basal metabolic cost was lower, and implied a total investment for the 10-day experimental period of 207 J indiv^-1^ (137 h) (Table 1).

**Table 1:** Summary of the metabolic costs (basal and actively feeding), energy incomes from prey ingestion, and energy gains obtained by the predator from each of the diets over the 10-day experimental period.

Diet characteristics	Prey species	Basal metabolism (J)	Active metabolism (J)	Prey energy (J)	Energy profit (J)
Monodiet	*P*. *purpuratus*	266 (176 h)	243 (64 h)	2532	2023
	*S*. *algosus*	216 (143 h)	455 (97 h)	4146	3475
Mixed diet	*P*. *purpuratus* + *S*. *algosus*	207 (137 h)	494 (102h)	4352	3651

Total times of basal or active metabolism are shown in brackets.

The energetic investment of the active metabolism during the attack and ingestion of prey over the 10-day period was 243 J indiv^-1^ (64 h spent attacking and ingesting prey) and 455 J indiv^-1^ (97 h) for *P*. *purpuratus* and *S*. *algosus* monodiets, respectively. When offered a mixed diet, the energetic investment of the active metabolism during the attack-ingestion of prey over the 10-day period, was even higher, at 494 J indiv^-1^ (102 h) (Table 1).

#### Energetic income

The energetic incomes were related to the energy content of the tissues consumed from each prey by the predator and to the number of prey consumed during the 10-day experimental period. Those incomes were almost twice as large for *S*. *algosus* (4146 J indiv^-1^) as they were for *P*. *purpuratus* (2532 J indiv^-1^) monodiets. Complementarily, when offered a mixed diet the energetic incomes were 4352 J indiv^-1^ during the same 10-day period.

#### Profit

Considering the costs and incomes noted above, the net energetic gain for the predators over the 10-day experimental period was 2023 J indiv^-1^ for the *P*. *purpuratus* monodiet and 3475 J indiv^-1^ for the *S*. *algosus* monodiet. The net energetic gain when individual gastropods were offered a mixed diet was 3651 J indiv^-1^ over the 10-day period (Table 1).

Spatial distribution of the attacks on prey shell valves and the compressive force required to break the prey valves.

**Fig 8:**
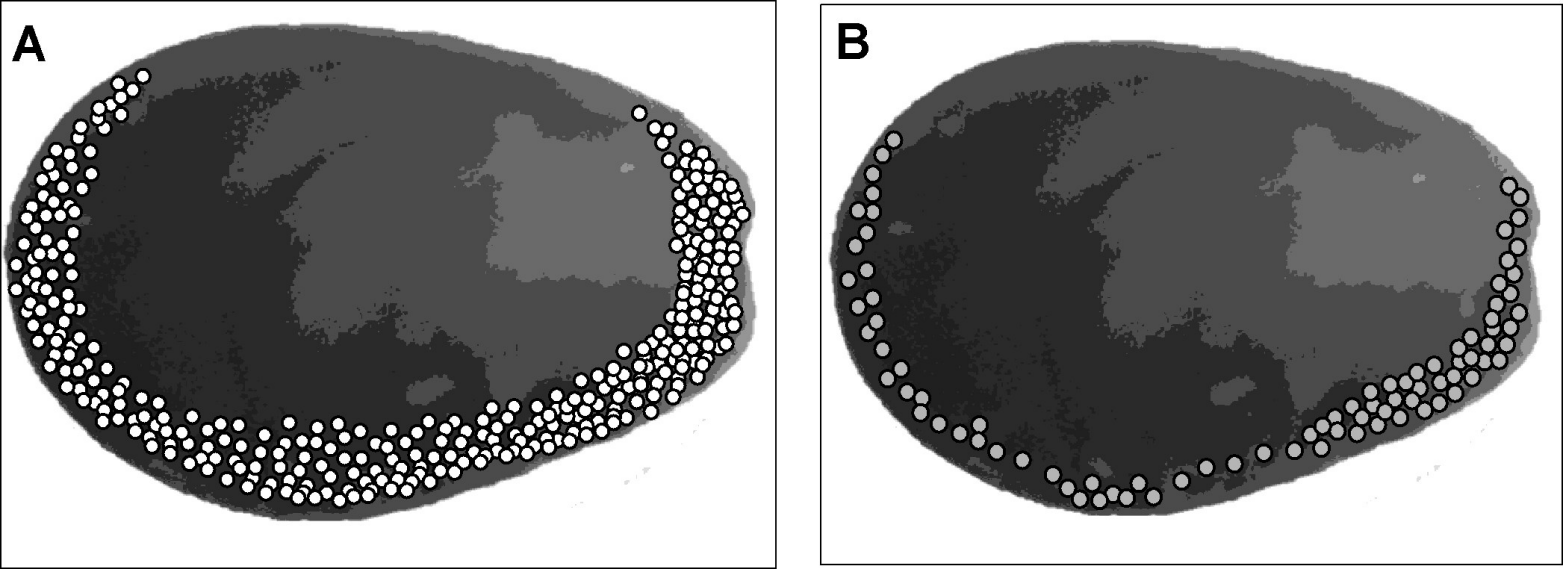
A) Spatial distribution of the attacks of the gastropod *A*. *monodon* on *S*. *algosus* (shell lengths of consumed prey varied between 13 and 25 mm); n total = 340 attacked preys and B) on *P*. *purpuratus* (shell lengths of consumed prey varied between 12 and 22 mm). n total = 88 attacked preys.

Attacks on both prey species were concentrated on the shell valve edges, in particular in the umbo, the ventral, and the posterior shell regions; no attacks were ever made in the central or dorsal regions of the valves (Fig 8A and 8B). Considering the analysis of the force required to break the valves, the amount of force needed to break different regions of the prey shell valves was significantly different for the different shell regions for both mussel prey species (ANCOVA, F_1,195_ = 223.29, *P* < 0.001).

**Fig 9:**
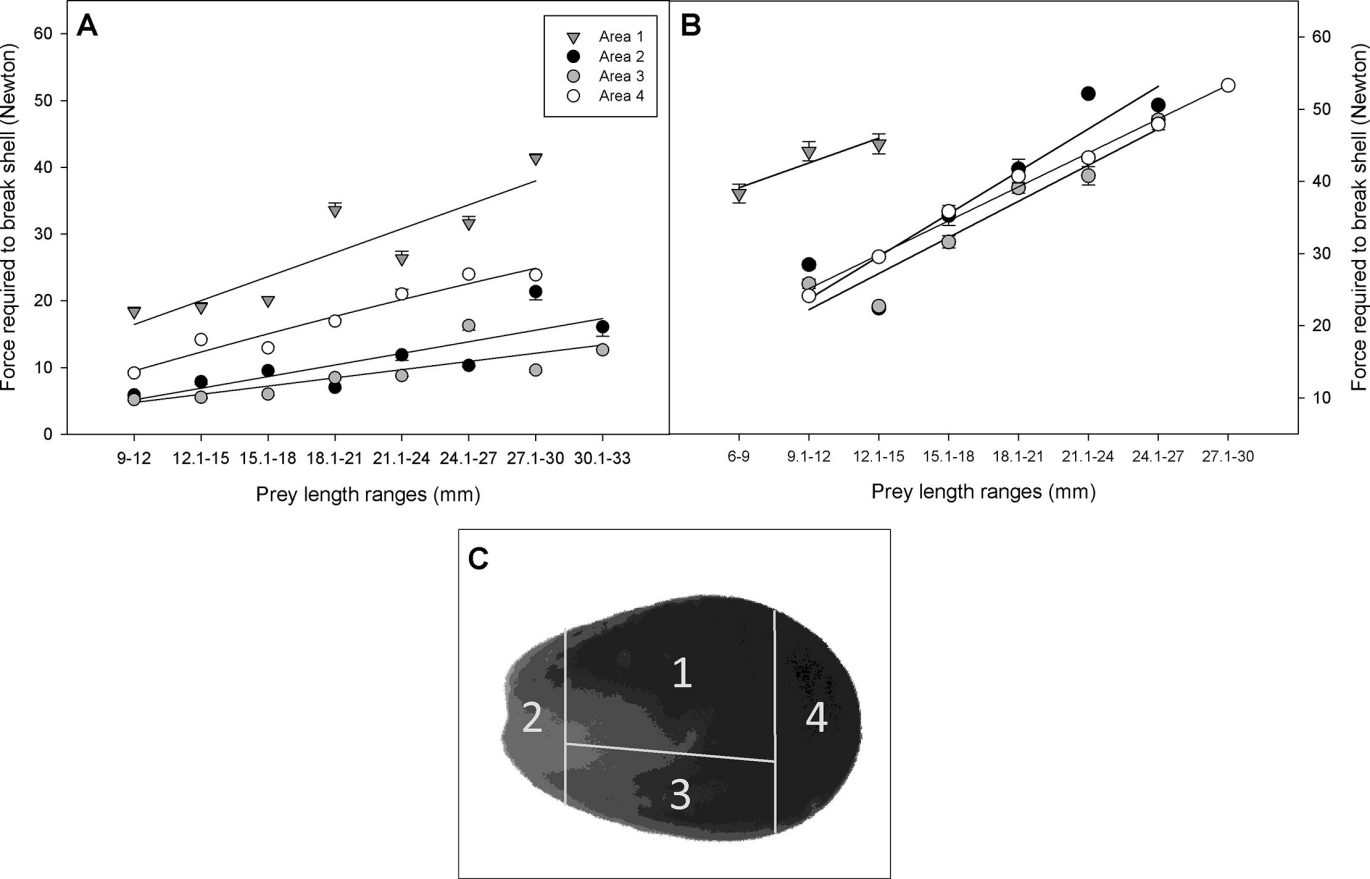
A) Compressive force required to break the prey valves of *S*. *algosus* for each valve area attacked in relation to prey size (mean ± SE). Area 1: y = 3.5851x + 12.883; r^2^ = 0.79. Area 2: y = 1.7405x + 3.441: r^2^ = 0.68. Area 3: y = 1.232x + 3.553; r^2^ = 0.63. Area 4: y = 2.5622x + 7.221; r^2^ = 0.94; n total = 120. B) Compressive force required to break the prey valves of *P*. *purpuratus* for each valve area attacked in relation to prey size (mean ± SE). Area 1: y = 3.435x + 35.693; r^2^ = 0.86. Area 2: y = 5.8873x + 11.949; r^2^ = 0.85. Area 3: y = 5.0072x + 12.22; r^2^ = 0.91. Area 4: y = 4.7345x + 15.625; r^2^ = 0.99; total n = 76. C) Scheme of attack areas: Area 1 = central area of the shell valve, area 2 = posterior area (umbo), area 3 = mid ventral area, area 4 = anterior area.

In *S*. *algosus*, the shells of larger bivalves required significantly more force to break (Fig 9A; ANCOVA, F_1, 117_ = 73.8, *P* < 0.000). Also, a significant relationship was observed in the forces required to break the valves between areas of the valves (Fig 9A; ANCOVA, F_3, 117_ = 65.41, *P* < 0.000. Areas 1 (center of the valve) and 4 (ventral posterior side of the valve) required the highest forces to break, compared to the other areas (Fig 9A, Tukey HSD test, *P* < 0.05). Meanwhile, areas 2 (anterior of the valve, umbonal area) and 3 (ventral-central side of the valve) required the lowest forces for breaking, and showed no significant differences between them (Fig 9A, Tukey test, *P* > 0.05).

In *P*. *purpuratus*, the shells of larger bivalves also required more force to break (Fig 9B; ANCOVA, F_1,71_ = 64.1, *P* < 0.0001). Moreover, the forces required to break the shell valves differed significantly between the different shell valve areas (Fig 9A; ANCOVA, F_3,71_ = 10.9, *P* < 0.0001). The central area (area 1) required the highest force to be broken (Tukey HSD test *P* < 0.05) compared to the other peripheral areas of the valves (areas 2, 3 and 4). No significant differences were observed in the forces required to break the shells between areas 2, 3 and 4 (Tukey HSD test *P* > 0.05).

## Discussion

### Prey selection and attack rate

The high costs of feeding for predator species are determined mainly by the difficulties of finding, handling, attacking and ingesting the prey [11, 35, 36]. These tasks may be particularly energetically expensive if the prey are not abundant and/or if they possess defense structures such as the shells of bivalves [10]. Predatory marine gastropods such as *A*. *monodon* that feed on bivalves need to optimize their feeding costs and energy incomes to successfully satisfy their metabolic requirements. In this study, energy benefits were maximized by selecting particular prey species, suggesting that prey selection can be explained by the energetic benefits obtained. Our results showed that the muricid gastropod *Acanthina monodon* consumed both mytilid species offered as prey, as observed in the field; however, the experiments in this study showed that when both species were available, the predator preferred *S*. *algosus* over *P*. *purpuratus* by a factor of 10, eating *S*. *algosus* in 90% of the events, and *P*. *purpuratus* in only 9% of the events. This distinct preference appears to be explained not by differences in the meat content of the two prey species, but basically by the higher energetic profit obtained when consuming *S*.*algosus* rather than the other prey species. However, this higher energetic difference did not result for the ingestion of one mussel of each prey species, but the result of consuming a larger number of *S*. *algosus* individuals per unit time. In a *P*. *purpuratus* monodiet scenario, the attack frequency (number of prey consumed by the predator per unit time) corresponded only to 48% compared to the number of a *S*. *algosus* individuals consumed in a monodiet of this mussel species. In studies with other invertebrates, such as the crab *Eriocheir sinensis*, prey selection also followed patterns that maximized net energy gain, which is partially explained by a much shorter handling time of the preferred prey compared to other available species [35]. Something similar was reported for the gastropod *Thais deltoidea*, a species that probably optimizes the energetic gain due to reduced handling and consumption times when preying on smaller preys [10]. Thus, the preference of one prey species over another may be defined by certain morphological characteristics, such as shell thickness [17, 37, 38]. Nonetheless, in generalist consumers the preference of one prey species over others is not only related to direct energetic gain, but also to the sizes of available prey, to nutritional components obtained from the prey, and to the handling times that define predatory costs [16].

Spatial distribution of the attacks on prey shell valves and the compressive forces required to break the shells.

Several researchers have reported the occurrence of a sort of ‘arms race’ between prey defenses vs. predatory modalities and spatial distribution of gastropod attacks [34, 39–42]. In our experiments, whenever the attacks of *A*. *monodon* left visible marks on the prey’s shell valves, these were always along the shell periphery (e.g. umbo, ventral edge and posterior region). No attacks in the center or on the dorsal peripheral edge of the valves were observed. The species *Chicoreus dilectus* also drills at the edges of the prey’s shell (periphery of the valves) and not in the center. This could be the result of an optimization of the attack energetic cost, as drilling located in the center required 3 times as much time as required by peripheral attempts, probably explained by differences in shell thickness between both sites [40]. An absence of attacks in the areas of major thickness/resistance of the prey’s shell valve was also reported for *A*. *monodon* when it was attacking other bivalve prey species [28]. Many other predatory gastropods, in particular muricids and some naticids, are also known to target particularly weak areas of their prey’s shells [11, 34, 40, 42, 43]. Recently, ontogenetic shifts in the attack strategies of *A*. *monodon* were reported, and explained by the gradual development of effective attack structures [27]. Future studies should focus on the energetic basis underneath such shifts that might be explained by varying balances between the energetic costs and incomes of each attack. Our results with *A*. *monodon* were obtained in the laboratory under controlled experimental conditions, where mussels were not in clumps or aggregations as they are in natural conditions in the field. This natural characteristic of clumping with neighboring mussels when adhered to the rocks, can effectively reduce the likelihood of predation [44], reducing the amount of shell surface exposure, in particular the weaker peripheral areas of the valves. Thus, the pattern of spatial distribution of attacks reported in this study may be modified in the field, something that can be investigated in future studies. In nature, the byssal threads produced by mussels may act as a defense mechanism against predators, in particular by strongly attaching the bivalves to the substrate, as well as to other mussels in the large multilayer mytilid clumps that are common along the intertidal rocky shore [45]. That the mytilid byssus can serve as an entrapment defense weapon or tool to hold and immobilize the predator, has been recorded in other mytilid species [46], but was not in the present research.

In general, our research showed that the shell valves of *P*. *purpuratus* required a greater force to be broken compared than those of *S*. *algosus*. This coincides with previous reports that the shell valves of *P*. *purpuratus* are thicker than those of *S*. *algosus* [17], which also explains why the attack time by *A*. *monodon* was shorter than that observed for *S*. *algosus*.

These results suggest that prey selection by *A*. *monodon* is not only defined by the energy contained in the prey tissues ingested, but is also related to the reduced costs of finding, handling, and attacking the prey, related to the shell valves’ hardness, the attack modality used, and the duration of the predatory process. In particular, the time expended in the attack process may relate to the risks of being washed out by wave exposure and/or being predated in the intertidal zone [47]. Experiments conducted by Grey et al. [38] showed that the naticid predator *Euspira lewisii* preferred to attack morphs of the bivalve prey *Protothaca staminea* that had thinner shell valves. Accordingly, the thicker valves of *Isognomon bicolor* compared to those of *Perna perna* reduced the drilling or chipping efficiency by whelks [48]. Also, although the predatory gastropod *Hexaplex trunculus* showed no preference for drilling either the left or right valves of *Mytilus galloprovincialis*, the drilling was usually done along the posterior margin of the shells [41]. Thus, the preference of *A*. *monodon* to prey on *S*. *algosus* appears to be related, at least partially, to their thinner shell valves, which required less force to be broken, and consequently to a shorter handling time and smaller cost. This is probably the major explanation for the very clear preferential selection of *S*. *algosus* over the sympatric mytilid species *P*. *purpuratus* by *A*. *monodon*.

### Soft tissue content/energy, handling ingestion of prey and resting time

Although partial consumption of prey has been reported for some gastropod species, with individuals possibly selecting particular organs that represent higher energetic incomes or that are more easily ingested [39, 41], the predator *A*. *monodon* consumed the prey tissues completely, as has also been observed in a number of other predatory gastropods (e.g. *Nucella lapillus*, [47]; *Adelomelon ancilla* [49].

In our study, *Acanthina monodon* needed a mean attack-ingestion time of 18 h when preying on *S*. *algosus* and and 25 h when preying on *P*. *purpuratus*. Those values showed wide fluctuations of approximately 65% of the mean time for each respective species. Strong variations in attack times have also been identified for some other carnivorous gastropods, such as *Nucella lapillus* [50] and *Hexaplex trunculus* [51]. In some cases, attack-ingestion time may be related to the size of the attacked prey. This is evident in the < 4h handling time of *Stramonita haemastoma* preying on small *Brachidontes pharaonis*, while preying on large individuals required up to 16 h (9.2 ± 3.6 h [52]. Similarly, the average prey-handling times (on *Mytilus galloprovincialis*) for small, medium, and large *Hexaplex trunculus* varied between 30.0 and 78.5h [41]. Moreover, these attack-ingestion times may vary depending on the mechanism the predators use to attack the prey as observed in *Hexaplex trunculus* and *Nucella lapillus* [41, 47]. In the present study, under similar conditions of temperature, gastropod sizes, and prey sizes, a significant difference in handling time was recorded for the two different prey species, which was related to differences in shell hardness between species. In contrast, resting times were higher when the predators were feeding on *P*. *purpuratus*, suggesting that besides investing more time in the attacks, the predator also needed longer resting times before initiating a second attack. The duration of resting times could also be influenced by factors such as prey type, prey size, or periods of harsh environmental conditions, as reported for the muricid *Nucella lapillus* [53].

### Oxygen consumption rate and metabolic costs

Basal oxygen consumption rates (OCR’s) are commonly less than consumption rates recorded during various animal activities [54]. For *A*. *monodon*, active OCR was 3.2 times the basal rate when preying on *S*. *algosus* and 2.5 times the basal OCR, when preying on *P*. *purpuratus*. Similar changes in OCR between resting periods and periods of activity have been reported for the carnivorous gastropod *Thais (N) lapillus* when comparing feeding activity with the period between meals [54].

Although the effective attack times seen in our study were 26% higher when *A*. *monodon* attacked *P*. *purpuratus* than when they attacked *S*. *algosus*, the OCR per unit time was higher when the predator was feeding on *S*. *algosus*, suggesting a higher metabolic investment during this activity. Nonetheless, when OCR is considered in relation to the effective attack-ingestion times for each prey, the attacks on *S*. *algosus* appear more profitable for the predator. This is explained by the shorter attack time, higher attack frequency, and consequently shorter resting times (between two consecutive attacks) when preying on *S*. *algosus*.

### Energetic summary

Our results suggest that the preference of *A*. *monodon* for preying on *S*. *algosus* over *P*. *purpuratus* is explained by the size of the overall energetic profit, despite the fact that the energetic cost of predation (per unit time) on the mussel *S*. *algosus* was higher, and the weight of the soft tissues as well as the energetic content (per gram) was lower, in this prey species. In contrast, the time invested in attack-ingestion of each individual was also shorter. Also, a shorter resting time between two consecutive attacks on *S*. *algosus* resulted in the energetic income per unit time (in this 10-day study) being significantly greater when *A*. *monodon* was consuming *S*. *algosus* than when it was consuming *P*. *purpuratus*. This result supports the idea that prey selection is associated with the balance between energetic investment and profit per unit time.

It is remarkable that at our study site, although *S*. *algosus* is an intertidal species with yearly events of high recruitment, the juveniles and adults have a clear seasonal presence that makes this species intermittent as a potential prey for *A*. *monodon*. This coincides with reports that along the central coasts of Chile, the dominant competitor *P*. *purpuratus* is found in greater abundance along most shores, while the subordinate species *S*. *algosus*, although commonly found, is persistently abundant at only a smaller set of locations along these shores [55]. Moreover, *S*. *algosus* has been described as a weak competitor with respect to *P*. *purpuratus*, related to a certain capacity for it to occupy sites with less competition for space in the intertidal, and with a lower attachment strength for both juveniles and adults [56]. At our studied site, *A*. *monodon* can access large numbers of *P*. *purpuratus* individuals throughout the year (Salas-Yanquin, Obs. Pers.), which allows the predator to survive the periods of low *S*. *algosus* presence or abundance. This is related to the fact that *S*. *algosus* is mainly observed only between January and March, and occasionally until June [17]. In any case, it seems that any predators inhabiting the rocky intertidal shore may potentially select between different available prey species according to their energetic quality [16, 57], but not necessarily to their abundance [58].

In our estimations of energetic costs and gains, we assumed that mussels were always completely ingested, as they were in our laboratory studies. However, this assumption may not be valid in the field, where wave exposure and potential competition with conspecifics could influence the gastropod to abandon its prey before consumption has been completed. Thus, in the field, the consumption and absorption efficiencies may vary between consumed prey individuals. Future studies should consider the effective use of the predated mussels, the effective assimilation of the nutrients ingested, and the potential changes in physiological response as a function of seasonality. In our study, the energetic cost estimations based in respiratory rates assumed that oxygen consumption measured in the prey/predator system were only due to the predator’s metabolic respiration. This may be correct: the sole presence of predator exudate has been found to generate a significant reduction of OCR in *P*. *purpuratus’* (Riedemann, Pers. Comm.) or even the complete closure of its shell valves, which can then force the activation of anaerobic metabolism [59] or generate high oxygen debt to the organisms [60]. In the same way, studies of the prey valve aperture and closure dynamics have shown that the physical contact of *A*. *monodon* with *P*. *purpuratus* causes the mussels to close their shell valves, significantly reducing rates of oxygen consumption (Riedemann, Pers. Comm). Future research should determine if, under these conditions, the mussels effectively cease external oxygen consumption, and instead begin to use the oxygen dissolved in the fluids trapped inside the pallial cavity after valve closure [61]. Although no information is available for *S*. *algosus*, the same questions are valid and should also be investigated.

Our results showed that the muricid gastropod *A*. *monodon* offered a choice of two sympatric mytilid species preferentially consumed mainly *S*. *algosus* over *P*. *purpuratus*. Preying on *S*. *algosus* required a lesser force to break the shell valves and a shorter time for consuming the prey’s tissues. Also, although each *S*. *algosus* individual generated smaller energetic incomes than *P*. *purpuratus* (both mussels showed similar energetic content per gram), the sum of the whole consumption per unit time (considering a shorter attack and resting time for *S*. *algosus* compared to *P*. *purpuratus*) makes *S*. *algosus* a more profitable prey to be consumed, when it is available intertidally. As expressed in prey selection models, our results indicate that prey are selected based on their higher energetic quality, while energetically suboptimal prey species are only ingested when optimal species are not available in the field [62, 63]. Murdoch [64] and Hughes & Croy [65] reported that prey availability may determine predator behavior, which may explain the high frequency of consumption of the mytilid *S*. *algosus* over *P*. *purpuratus*, particularly when the abundance of *S*. *algosus* is seasonal [17], and *P*. *purpuratus* is available during the whole year.

In summary, *A*. *monodon* is a carnivorous gastropod that feeds on both of the mussel species used in our study. Although *P*. *purpuratus* was not the preferred prey in this study, the energetic balance was positive, and the abundance of this species was constantly higher in the field throughout the year. Thus, the preferred selection of *S*. *algosus* by *A*. *monodon* is apparently related to a higher energetic balance compared to *P*. *purpuratus*. This is associated with a greater energetic profit per unit of time, rather than to the total amount of energy obtained from the consumed prey.

## Supporting information

S1 Appendix Data set on different physiological variables involved in the prey selection between two mytilds species by the gastropod *Acanthina monodon*. (XLSX)
